# “Skip the Small Talk” Virtual Event Intended to Promote Social Connection During a Global Pandemic: Online Survey Study

**DOI:** 10.2196/28002

**Published:** 2021-09-23

**Authors:** Jasmine Mote, Kathryn Gill, Daniel Fulford

**Affiliations:** 1 Department of Occupational Therapy Tufts University Medford, MA United States; 2 Department of Occupational Therapy Sargent College of Health and Rehabilitation Sciences Boston University Boston, MA United States

**Keywords:** COVID-19, depression, digital group, loneliness, social connection, virtual social interaction, community, mental health, connection, virtual health

## Abstract

**Background:**

Social distancing measures meant to prevent the spread of COVID-19 in the past year have exacerbated loneliness and depression in the United States. While virtual tools exist to improve social connections, there have been limited attempts to assess community-based, virtual methods to promote new social connections.

**Objective:**

In this proof-of-concept study, we examined the extent to which Skip the Small Talk (STST)—a business dedicated to hosting events to facilitate structured, vulnerable conversations between strangers—helped reduce loneliness in a virtual format in the early months of the 2020 COVID-19 pandemic. We predicted that participants who attended STST virtual events would show a reduction in loneliness, improvement in positive affect, and reduction in negative affect after attending an event. We were also interested in exploring the role of depression symptoms on these results as well as the types of goals participants accomplished by attending STST events.

**Methods:**

Adult participants who registered for an STST virtual event between March 25 and June 30, 2020, completed a survey before attending the event (pre-event survey; N=64) and a separate survey after attending the event (postevent survey; n=25). Participants reported on their depression symptoms, loneliness, and positive and negative affect. Additionally, participants reported the goals they wished to accomplish as well as those they actually accomplished by attending the STST event.

**Results:**

The four most cited goals that participants hoped to accomplish before attending the STST event included the following: “to make new friends,” “to have deeper/better conversations with other people,” “to feel less lonely,” and “to practice social skills.” A total of 34% (20/58) of participants who completed the pre-event survey reported depression symptoms that indicated a high risk of a major depressive episode in the preceding 2 weeks. Of the 25 participants who completed the pre- and postevent surveys, participants reported a significant reduction in loneliness (*P*=.03, Cohen *d*=0.48) and negative affect (*P*<.001, Cohen *d*=1.52) after attending the STST event compared to before the event. Additionally, depressive symptoms were significantly positively correlated with change in negative affect (*P*=.03), suggesting that the higher the depression score was prior to attending the STST event, the higher the reduction in negative affect was following the event. Finally, 100% of the participants who wished to reduce their loneliness (11/11) or feel less socially anxious (5/5) prior to attending the STST event reported that they accomplished those goals after the event.

**Conclusions:**

Our preliminary assessment suggests that the virtual format of STST was helpful for reducing loneliness and negative affect for participants, including those experiencing depression symptoms, during the COVID-19 pandemic. While encouraging, additional research is necessary to demonstrate whether STST has benefits when compared to other social events and interventions and whether such benefits persist beyond the events themselves.

## Introduction

Prior to the COVID-19 global pandemic, caused by SARS-CoV-2, public health researchers in the United States were concerned with a different social health epidemic: loneliness. A 2019 national survey found that 61% of US adults reported significantly high trait loneliness, a 7% increase from the previous year [1]. Loneliness, or the aversive experience of one’s social needs not being met [2,3], is a public health concern given its association with heightened risk of cardiovascular disease, mental health difficulties, and early mortality [4]. Recently, social distancing measures to combat the spread of COVID-19 have led to a reduction of in-person opportunities for both mental health support and general socialization [5]. Reductions in frequency of social interactions and increases in loneliness are both associated with increases in depression symptoms [6], a mental health concern that has increased in prevalence during the pandemic [7]. Researchers have also found a reduction in social participation [8] and poorer sleep [9] since the onset of the pandemic, two negative outcomes that are also related to loneliness. Specific to loneliness, Killgore and colleagues [10] found an increase in loneliness postpandemic compared to prepandemic in a sample of participants proportionally representative of the United States. Loneliness rates throughout the pandemic have been associated with depression symptoms and suicidal ideation, with some suggesting that these concerns have worsened over the course of the past year [10,11].

While virtual tools to improve social activity and loneliness existed prior to the pandemic, these measures have seen an increase in accessibility and prioritization postpandemic. Social media, mobile health tools (eg, smartphone app–based interventions), web-based platforms, and video tools (eg, Zoom) have all been studied as means of improving social connection [12]. For example, Shapira and colleagues [13] found that small group sessions hosted via Zoom where participants learned and practiced cognitive and behavioral coping techniques were effective at reducing feelings of loneliness and depression during the pandemic. However, not all means of connecting with others virtually have shown reductions in loneliness, with one recent study failing to find an association between reductions in loneliness and frequency of video calls with friends, acquaintances, family, or romantic partners [14]. Using virtual methods to teach new skills and/or foster new social connections may be particularly helpful in addressing loneliness during this unique time in history.

Skip the Small Talk (STST) is a formerly Boston-based business that traditionally hosts in-person social events intended to help people “get closer, faster.” STST’s approach is informed by research that suggests that strong social connection and intimacy can be promoted between strangers through structured, open-ended questions that involve self-disclosure [15]. During these events, strangers have one-on-one and group conversations and are encouraged to be vulnerable and open with one another. This includes framing discussions around questions that have been cited in the popular press as the “36 questions that lead to love,” among other questions that prompt self-disclosure and intimacy [16]. STST has hosted in-person public and private events (eg, college orientations and corporate events) throughout the United States. At the onset of the 2020 COVID-19 global pandemic, STST translated their events into a virtual format.

In this proof-of-concept study, we examined the extent to which virtual STST, delivered during the early months of the COVID-19 pandemic, reduced loneliness and improved social connection for attendees. We recruited people who were interested in attending the virtual events to complete pre- and postevent surveys to assess these concerns. We predicted that participants who attended STST virtually would report a reduction in loneliness, improvement in positive affect, and reduction in negative affect after the event compared to before attending the event. We also examined the extent to which depression symptoms influenced these results given the relationship between loneliness and depression symptoms, particularly during the pandemic [10,11]. We were also interested in exploring the following: (1) the extent to which the pandemic was influencing people’s decisions to attend STST, (2) the extent to which the pandemic was affecting people’s social activities and feelings of connection with others, (3) the types of social goals people wanted to accomplish by attending STST, and (4) whether STST addressed participants’ anxiety and stress related to the pandemic.

## Methods

### Recruitment

Participants were a convenience sample of adults over the age of 18 years who registered for an STST virtual event between March 25 and June 30, 2020, and who agreed to complete the pre- or postevent survey. To incentivize completion of both surveys, participants who completed both the pre- and postevent surveys were entered into a raffle to win one of two US $50 Amazon gift cards or a gift card for a local business of their choice. A total of 64 participants completed portions of the pre-event survey, and 25 of those participants completed a portion of the postevent survey after the event, with a minimum duration of 90 minutes in between completing both surveys (ie, the duration of the STST event). Participants were excluded from postevent survey analysis if they completed the pre- and postevent surveys back-to-back (eg, within a few minutes of each other or completion of the pre-event survey after the postevent survey). See Table 1 for demographic information.

### Skip the Small Talk Virtual Event Format

People interested in attending an event signed up through the STST website. Virtual events cost participants US $10 until June 12, 2020, when participants followed a “pay what you wish” fee schedule to improve access to the events during the pandemic; the average payment per participant per event from June 12 to 30 was US $4. The events lasted approximately 90 minutes and were facilitated and structured by a member of the STST team. This time period included the beginning of the event, when all participants were provided with event details and expectations. Then, participants completed two dyadic conversations where they were paired with a conversational partner at random and engaged in a brief conversation structured around answering a question designed to promote intimacy and self-disclosure. These dyadic conversations lasted approximately 17 minutes each, with participants taking turns listening, responding to the question, and engaging in back-and-forth conversation. After the dyadic conversations, participants then engaged in a 12-minute group conversation among 3 or 4 other participants where they responded freely to another question. Finally, at the end of each event, participants engaged in a 3-minute dyadic conversation with another participant where they answered the question, “What’s at least one thing you’d like to take with you from this event?” The remaining time for each event consisted of structured breaks in between conversations and reminders for participants to share more than they might usually share in their everyday lives, have compassion for others, notice their emotions, and reflect on their conversations.

### Self-report Scales

#### Skip the Small Talk Goals

Participants reported on a selection of one or more potential goals they either wished to accomplish (on the pre-event survey) or accomplished (on the postevent survey) by attending STST. The goals included the following: “to make new friends,” “to meet a potential romantic partner,” “to practice being vulnerable”, “to practice social skills/get better at talking with people,” “to feel less socially anxious,” “to feel less lonely,” “to have deeper/better conversations with other people,” or “other” (write-in option). Participants could select multiple goals.

#### Depression

A subset of participants (58/64, 91%) reported symptoms of depression over the past 2 weeks on the pre-event survey by completing the 8-item Patient Health Questionnaire (PHQ-8) [17]. Participants reported whether they felt a specific symptom (eg, “Little interest or pleasure in doing things”) on a scale from 0 (“not at all”) to 3 (“nearly every day”). A total sum score was calculated, with higher scores reflecting more severe depression symptoms and a score greater than or equal to 10 suggesting heightened risk for meeting criteria for a current major depressive episode [17].

#### Loneliness

A subset of participants completed the 8-item UCLA (University of California, Los Angeles) Loneliness Scale (UCLA-LS-8) as an assessment of loneliness during the pre-event (61/64, 95%) and postevent surveys (24/25, 96%) [18]. Participants reported how applicable various descriptive statements were to them (eg, “I lack companionship”) on a scale from 1 (“I never feel this way”) to 4 (“I often feel this way”). A total sum score was calculated, with higher scores reflecting more loneliness.

#### Positive and Negative Affect

A subset of participants completed assessments of positive and negative affect for the pre-event (61/64, 95%) and postevent surveys (25/25, 100%). Participants reported how often they felt a series of positive and negative emotions on a 5-point Likert scale from 1 (“never”) to 5 (“all the time”). Participants either reported on how much they felt these emotions over the past week (pre-event survey) or at the present moment (postevent survey). Positive emotions included happy, excited, calm, cheerful, and relaxed. These scores were averaged to create a positive affect score. Negative emotions included angry, bored, lonely, anxious, sad, and sluggish. These scores were averaged to create a negative affect score. Higher scores reflect higher frequency of positive or negative emotions felt.

### Procedures

#### Overview

People who signed up for the virtual STST events received an email with the pre-event survey link and were instructed to complete the survey prior to attending the virtual event. Immediately following the virtual event, event participants were emailed the link to the postevent survey and reminded to complete it by the STST event coordinator. In order to match pre-event and postevent survey respondents, participants were instructed to enter a unique phrase, screen name, or email address for each survey. This information was deleted immediately after pre- and postevent survey responses were matched. Both surveys were opened and administered using Qualtrics.

#### Pre-event Survey

Participants reported standard demographic information, including age, gender, ethnicity, and marital or relationship status. Participants also reported whether they had ever attended an STST event before, either in person or virtually; the date of the virtual event they planned on attending; and the goals they planned to accomplish (see the Skip the Small Talk Goals section). Participants also completed the depression (PHQ-8), loneliness (UCLA-LS-8), and positive and negative affect questions. Additionally, participants were asked to respond on a 5-point Likert scale, from 1 (“not at all”) to 5 (“a great deal”), to the following questions related to STST and the COVID-19 pandemic: “How much are you looking forward to the Skip the Small Talk event?” “How anxious/stressed have you felt in the past week due to the COVID-19/coronavirus pandemic?” “Have you been spending less time with people in the past week due to the COVID-19/coronavirus pandemic?” and “Have you felt less connected to people in the past week due to the COVID-19/coronavirus pandemic?” Finally, participants were asked, “Was your decision to attend the STST event directly related to wanting to find ways to connect with others during the COVID-19/coronavirus pandemic?” Response options included “yes,” “no,” or “maybe.”

#### Postevent Survey

Participants reported the date of the virtual event they attended. They also reported on the goals that they felt they accomplished by attending the event (see the Skip the Small Talk Goals section). Then, they completed the loneliness questionnaire again (UCLA-LS-8) as well as the positive and negative affect questions. Participants were also asked to respond on a 5-point Likert scale, from 1 (“not at all”) to 5 (“a great deal”), to the following questions related to STST and the COVID-19 pandemic: “How much did you enjoy the Skip the Small Talk event?” “Did this event help you feel less anxious/stressed during the COVID-19/coronavirus pandemic?” and “Did this event help you feel more connected to others during the COVID-19/coronavirus pandemic?”

### Statistical Analysis

Pre- and postevent survey responses were matched using the date of the event attended and the unique phrase, screen name, or email address that participants reported. Duplicates were removed prior to analysis. Available case analysis was used for all analyses. Chi-square and independent-samples *t* tests were computed to assess differences in history of attending STST events and demographic variables between participants who only completed the pre-event survey versus those who completed both the pre- and postevent surveys. Paired-samples *t* tests and Pearson correlations were computed to assess the differences in pre- versus postevent survey responses (eg, positive and negative affect) and the relationship between pre-event survey depression scores and other outcomes, respectively. Effect sizes (Cohen *d*) were computed where appropriate.

## Results

### Demographics and Other General Information

Participants who completed the surveys ranged in age from 22 to 58 years and predominantly identified as White, women, and single (see Table 1). They had predominantly either never attended a virtual or in-person STST event before or had only attended an in-person STST event in the past.

**Table 1:** Summary of demographic information and Skip the Small Talk (STST) exposure.

Demographics and STST exposure			Pre-event respondents (N=64)	Pre- and postevent respondents (n=25)
**Age in years**				
	Mean (SD)	32.83 (8.04)		33.64 (8.44)
	Range	22-58		23-58
**Gender, n (%)**				
	Men	13 (20)		3 (12)
	Women	47 (73)		22 (88)
	Transgender men	1 (2)		0 (0)
	Nonbinary or 3rd gender	2 (3)		0 (0)
	Did not report gender	1 (3)		0 (0)
**Relationship status, n (%)**				
	Married or in a relationship	18 (28)		9 (36)
	Divorced or separated	4 (6)		1 (4)
	Single	41 (64)		15 (60)
	Self-described: polyamorous	1 (2)		0 (0)
**Ethnicity, n (%)**				
	White	40 (63)		14 (56)
	Hispanic or Latinx	7 (11)		4 (16)
	Asian or Asian American	7 (11)		1 (4)
	Other or multiple ethnicities	8 (13)		5 (20)
	Did not report ethnicity	2 (3)		1 (3)
**STST exposure, n (%)**				
	Never attended STST before	34 (53)		11 (44)
	Only attended in-person STST before	25 (39)		10 (40)
	Only attended virtual STST before	3 (5)		3 (12)
	Attended both in-person and virtual STST	2 (3)		1 (4)

Participants, on average, reported subclinical depression symptoms; approximately one-third of participants (20/58, 34%) who completed the pre-event survey reported a score of 10 or above on the PHQ-8 (Table 2), which indicates a high risk of a major depressive episode in the 2 weeks prior to the event. Participants who completed the pre-event survey (61/64, 95%) reported a UCLA-LS-8 mean total score of 19.38 (SD 4.26).

Of the participants who completed both the pre- and postevent surveys (25/64, 39%), the majority completed both surveys within the same day (20/25, 80%), where 2 hours was the shortest duration between completing the two surveys. Participants who only completed the pre-event survey compared to those who completed the pre- and postevent surveys did not significantly differ in mean age (*t*_62_=0.64, *P*=.52), PHQ-8 total score (*t*_56_=1.09, *P*=.28), or UCLA-LS-8 total score (*t*_59_=0.94, *P*=.35). Groups also did not differ in proportion of gender (χ^2^_4_=5.1, *P*=.27) or race or ethnic background (χ^2^_5_=5.7, *P*=.34). Additionally, groups did not significantly differ in proportion of participants who had never attended an STST event (χ^2^_1_=1.4, *P*=.24), previously attended in-person STST events (χ^2^_1_=0.02, *P*=.90), or previously attended both in-person and virtual events (χ^2^_1_=0.1, *P*=.75). The only difference we found between groups was that all the participants who had attended a virtual STST event in the past (3/64, 5%) completed both the pre- and postevent surveys (χ^2^_1_=4.9, *P*=.03).

**Table 2:** Summary of depression scores using the PHQ-8.^a^

Measure and outcome	Pre-event respondents (n=58)	Pre- and postevent respondents (n=24)
PHQ-8 score (pre-event), mean (SD)	7.83 (4.64)	7.04 (3.80)
Participants reporting scores of 10 or above on the PHQ-8, n (%)	20 (34)	6 (25)

^a^PHQ-8: 8-item Patient Health Questionnaire.

### Responses to Skip the Small Talk and COVID-19 Questions

Table 3 shows the average responses to specific questions related to STST and the COVID-19 pandemic. The majority of questions were rated on a 5-point Likert scale from 1 (“not at all”) to 5 (“a great deal”). Participants who only completed the pre-event survey compared to those who completed the pre- and postevent surveys did not significantly differ in responses to any of the questions (all *P* values >.10). Overall, participants reported that they were generally feeling less connected with others and spending less time with others due to the COVID-19 pandemic. Over half of the participants reported that their decision to attend an STST event was directly related to a desire to find ways to connect with others during the pandemic. Additionally, participants reported a moderate amount of anxiety and stress related to the pandemic.

**Table 3:** Responses to questions about Skip the Small Talk (STST) and the COVID-19 pandemic.

Question^a^			Pre-event respondents (n=59)		Pre- and postevent respondents (n=25)		*P* value
How much are you looking forward to the STST event? (pre-event respondents, n=64), mean (SD)			3.48 (0.78)		3.68 (0.80)		.11
How much did you enjoy the STST event?, mean (SD)			N/A^b^		4.32 (0.80)		N/A
Have you felt less connected to people in the past week due to the COVID-19/coronavirus pandemic?, mean (SD)			3.41 (1.31)		3.12 (1.36)		.15
Have you been spending less time with people in the past week due to the COVID-19/coronavirus pandemic?, mean (SD)			3.73 (1.41)		3.60 (1.44)		.55
Did this event help you feel more connected to others during the COVID-19/coronavirus pandemic? (postevent respondents, n=24), mean (SD)			N/A		3.88 (0.95)		N/A
How anxious/stressed have you felt in the past week due to the COVID-19/coronavirus pandemic?, mean (SD)			3.29 (1.15)		3.32 (1.35)		.86
Did this event help you feel less anxious/stressed during the COVID-19/coronavirus pandemic? (postevent respondents, n=24), mean (SD)			N/A		3.25 (1.26)		N/A
**Was your decision to attend the STST event directly related to wanting to find ways to connect with others during the COVID-19/coronavirus pandemic?, n (%)**							
	Yes	38 (64)		16 (64)		.96	
	No	13 (22)		5 (20)		.75	

^a^The majority of questions were rated on a 5-point Likert scale from 1 (“not at all”) to 5 (“a great deal”).

^b^N/A: not applicable; these questions only applied after the event.

### Social Goals

Participants who completed the pre-event survey reported a mean of 3.75 (SD 1.69) goals that they wanted to accomplish by attending the STST event (Table 4). The four most frequently cited goals were as follows: “to make new friends,” “to have deeper/better conversations with other people,” “to feel less lonely,” and “to practice social skills/get better at talking with people.” Examples of “other” write-in responses included curiosity about using virtual platforms to connect with others, a desire to increase social contact, and specific goals unrelated to the ones listed (eg, looking for coworking partners).

**Table 4:** Summary of participant goals for attending Skip the Small Talk.

Goal	Pre-event respondents (N=64), n (%)	Pre- and postevent respondents (n=25), n (%)
Make new friends	52 (81)	20 (80)
Meet a potential romantic partner	27 (42)	11 (44)
Practice being vulnerable	20 (31)	10 (40)
Practice social skills/get better at talking with people	34 (53)	14 (56)
Feel less socially anxious	12 (19)	5 (20)
Feel less lonely	35 (55)	11 (44)
Have deeper/better conversations with other people	50 (78)	21 (84)
Other	10 (16)	5 (20)

### Comparing Pre- and Postevent Survey Responses

#### Accomplished Social Goals

Participants who completed both the pre- and postevent surveys reported on which social goals they accomplished from attending STST. Overall, participants reported a desire to accomplish a mean of 3.88 (SD 2.03) goals and accomplished a mean of 2.60 (SD 1.87) of those goals. Looking at individual goals for each participant, including the goals they planned to accomplish in the pre-event survey along with the goals they reported actually accomplishing in the postevent survey, the following results were found: 12 out of 20 participants (60%) reported accomplishing their goal “to make new friends,” 2 out of 11 participants (18%) reported accomplishing their goal “to meet a potential romantic partner,” 8 out of 10 participants (80%) reported accomplishing their goal “to practice being vulnerable,” 11 out of 14 participants (79%) reported accomplishing their goal “to practice social skills/get better at talking with people,” 5 out of 5 participants (100%) reported accomplishing their goal “to feel less socially anxious,” 11 out of 11 participants (100%) reported accomplishing their goal “to feel less lonely,” 15 out of 21 participants (71%) reported accomplishing their goal “to have deeper/better conversations with other people,” and 1 out of 5 participants (20%) reported accomplishing a self-described “other” goal. Outside of planned goals that were reported on the pre-event survey, participants also reported, on the postevent survey, accomplishing goals they did not originally report a desire to accomplish: 1 out of the 5 participants (20%) who did not plan to make new friends based on the pre-event survey reported that they accomplished that goal, 4 out of 15 participants (27%) reported that they practiced being vulnerable, 3 out of 20 participants (15%) reported that they felt less socially anxious, 5 out of 14 participants (36%) reported that they felt less lonely, and 1 out of 20 participants (5%) reported that they accomplished an unlisted “other” goal.

#### Changes in Loneliness, Affect, Enjoyment, and Anxiety or Stress

Participants reported a significant reduction in loneliness from before (mean 18.83, SD 3.90) to after (mean 17.58, SD 4.89) the STST event (*t*_23_=2.35, *P*=.03, Cohen *d*=0.48). They also reported more positive affect (mean 3.07, SD 0.89, to mean 2.74, SD 0.63; *t*_24_=2.03, *P*=.05, Cohen *d*=0.40) and less negative affect (mean 1.58, SD 0.66, to mean 2.58, SD 0.70; *t*_24_=7.60, *P*<.001, Cohen *d*=1.52) when comparing their affect the week before the event to their in-the-moment affect following the event. Participants reported enjoying STST after the event (mean 4.32, SD 0.80) more than they were looking forward to the event (mean 3.68, SD 0.80). We reverse-coded the item regarding anxiety and stress related to the COVID-19 pandemic in the past week during the pre-event survey so that higher numbers reflected lower anxiety (mean 2.67, SD 1.37). Participants reported that the STST event helped them feel less anxiety and stress related to the COVID-19 pandemic after the event (mean 3.25, SD 1.26). However, this was not significantly greater than the amount of anxiety and stress they reported prior to the event (*t*_23_=1.69, *P*=.11, Cohen *d*=0.34).

#### Correlations With Depression

We examined whether pre-event depressive symptoms were correlated with pre-event to postevent changes in loneliness (difference score, mean –1.25, SD 2.61), positive affect (mean 0.34, SD 0.83), and negative affect (mean –1.00, SD 0.66). We also examined whether depressive symptoms were correlated with total number of planned social goals accomplished by participants. Depression symptoms were significantly positively correlated with change in negative affect (*r*=–0.44, *P*=.03), suggesting that the higher the depression score was prior to the event, the higher the reduction in negative affect was following the event. Depression symptoms were not significantly correlated with change in loneliness (*r*=–0.19, *P*=.38), change in positive affect (*r*=–0.19, *P*=.37), or total number of social goals accomplished by participants (*r*=–0.24, *P*=.27).

## Discussion

### Principal Findings

This study serves as a proof-of-concept that a virtual version of STST, a community-based business dedicated to helping strangers quickly form intimate connections with one another, may contribute to reductions in loneliness and improvements in social connection during a global pandemic. Over half of the participants stated that their desire to attend the STST event was related to finding ways to connect with others during the pandemic. The most frequently cited goals for attending the event included to make new friends, have better conversations with others, and feel less lonely. All of the participants who wanted to reduce their loneliness and feel less socially anxious by attending the STST event reported that they achieved those goals; in addition, participants felt like they achieved those goals even when they did not plan to do so. Overall, participants reported enjoying the virtual event and that the event helped them feel less stress and anxiety from the COVID-19 pandemic. Further, as predicted, participants reported a reduction in both loneliness and negative affect after attending the event compared to before the event. We also found a significant correlation between depression symptoms and change in negative affect that suggested that the higher the participant’s depression score was pre-event, the higher the reduction in negative affect was following the event.

While encouraging, these results should be considered preliminary. We did not assess a comparison group (eg, unstructured virtual conversations with strangers) and, thus, are unable to conclude whether the structure of STST uniquely contributed to reductions in loneliness and negative affect over and above the experience of interacting with others. However, other studies conducted during the COVID-19 pandemic have shown that frequency of communication with close contacts by itself may not contribute to reductions in loneliness [14]. Thus, it remains imperative to continue to explore creative ways to combat social isolation–related distress and loneliness that do not rely solely on pre-existing relationships or in-person interactions.

### Strengths

Though speculative, the structure of STST may be particularly helpful for people experiencing loneliness, depression, or social dysfunction broadly. Throughout the event, participants are explicitly encouraged to be more intimate and vulnerable than they would be in their everyday lives, presenting the opportunity for participants to be a different version of themselves than they typically are in their other relationships. Further, the everyday pressures to be optimally socially skilled and to accurately read social cues to maintain the conversation as well as the cognitive load of deciding on whether to engage with the same person again in the future are all eliminated through the time-limited, structured nature of STST interactions. Taken together, the structure of STST may allow the positive qualities of the social experiences themselves, such as feeling connected and intimate with others, to shine through—the same qualities that are associated with less loneliness in everyday life [19]. Future research is necessary to clarify which aspects, if any, of STST (eg, explicit encouragement to be vulnerable, time-limited conversations, and lack of expectation to repeat interactions with the same person) may facilitate reductions in loneliness and improvements in social connection, particularly for individuals with social difficulties and/or mental health concerns, over and above unstructured social events.

STST events appeared to appeal to those experiencing mental health distress during the COVID-19 pandemic. Approximately one-third of participants who completed the pre-event survey reported depression symptoms that indicated a high risk for a major depressive episode in the preceding 2 weeks. Additionally, one of the most cited goals that participants reported wanting to accomplish through attending STST involved improving one’s social skills. Though speculative, events like STST may be beneficial for addressing broad distress during a global pandemic or in contexts where in-person interactions are unavailable (eg, hospital settings and connecting people internationally or those in remote or rural locations). Further research on the development, implementation, and evaluation of virtual events and interventions like STST is necessary to better understand the potential benefits they may have for those with mental health concerns.

### Limitations

Several limitations of this study are worth noting. The most significant limitation is the loss of approximately 60% (n=39) of pre-event respondents in completing the postevent survey. While we did not find many differences in characteristics between those who did and did not complete the postevent survey (eg, demographics), we cannot rule out the possibility that participants who enjoyed attending the events more may have been more likely to respond to the postevent survey, potentially biasing our results. The only difference we found between those who did versus did not complete the postevent survey was that all the participants who had a history of attending the virtual STST events (n=3) completed both surveys, suggesting that familiarity and preference for these events may have contributed to survey completion. Future researchers may wish to assess loneliness and feelings of social connection during, versus after, the STST events, or compare attendees with people who were interested in the event but did not end up attending, to reduce such biases. While we found a significant reduction in loneliness comparing the pre- and postevent survey responses, there may have been other factors that contributed to these changes that we did not assess (eg, acquiescence bias and other social interactions during the day). Further, our assessments of positive and negative affect compared different time periods: the pre-event survey assessed how participants felt in the past week prior to the STST event, whereas the postevent survey assessed how they felt in the moment after the event. Thus, it is difficult to conclude that STST specifically was responsible for these changes in affect. Additionally, because we were interested in a broad community-based sample of people experiencing distress during the COVID-19 pandemic, we did not explicitly recruit for participants who met a clinical threshold for a psychiatric disorder, nor did we collect participant psychiatric history. Thus, it is unclear whether reductions in loneliness or subjective feelings of addressing social anxiety would be the same magnitude in those with a history or currently experiencing clinical rates of depression or social anxiety disorder. Our sample was relatively young (mean age: pre-event 32.83 years; postevent 33.64 years), reflecting the typical age group that STST attracts. As older adults experience high rates of loneliness, particularly during the pandemic [20], recruiting for a broader age range and/or assessing businesses that are targeted to addressing loneliness in older adults is warranted. It remains an open research question as to whether attending multiple STST events helps contribute to reductions in loneliness, or whether attending these events leads to reductions in loneliness that are maintained over time. Further, we did not assess whether STST participants made friendships or other relationships that persisted beyond the event itself. As the pandemic enters its second year in the United States, understanding whether events like STST contribute to longer-lasting reductions in loneliness or improvements in social connections is a necessary next step.

### Conclusions

STST is a community-based business dedicated to hosting social events to improve social connection and foster intimacy between strangers. During the COVID-19 global pandemic in 2020, STST adapted its events to a virtual format. In a preliminary, open, pilot assessment, participants reported a significant reduction in loneliness and negative affect after attending the event and reported accomplishing numerous social goals, including addressing loneliness and social anxiety, through participating in STST. Additional research is necessary to demonstrate the benefits of STST over and above other social events and interventions. Future research should continue to assess synchronous, virtual events meant to promote social connection as one means of addressing the loneliness inherent in a society dealing with a pandemic that makes in-person engagement difficult.

## Abbreviations

PHQ-8: 8-item Patient Health Questionnaire
STST: Skip the Small Talk
UCLA-LS-8: 8-item UCLA (University of California, Los Angeles) Loneliness Scale
